# Situation and psychosocial well-being of older sisters to children with disabilities or chronic illnesses—the forgotten children?

**DOI:** 10.3402/qhw.v8i0.21755

**Published:** 2013-07-02

**Authors:** Ulrika Hallberg

It is estimated that about 18% of all children aged between 5 and 17 years suffer from some kind of disability or chronic illness (Pastor, Reuben, & Loeb, 2009). Most of these children live at home with their parents and siblings, which put high demands not only on the parents but also on the siblings (Abrams, 2009) who have been described as the “forgotten children” (Madan-Swain, Sexson Brown, & Ragab, 1993) and a “population at risk” (Hannah & Midlarsky, 1985). The number of research studies concerning these siblings has increased over the past decades even if most of the studies concern children with cognitive disabilities (Pit-ten Cate & Loots, 2000). It is not evident that one can generalize these results to children with physical disabilities or chronic illnesses since the demands of the different types of conditions put on the adjustment ability to their families are somewhat different (Sloper & Turner, 1993). Furthermore, most of this research studies the sibling's situation and well-being from the mother's perspective (Sari, Baser, & Turan, 2006; Stoneman, 2005) and siblings as a group and, thus, are not gender- or age-specific studies.

Today, it is well known that growing up with a sibling with a disability or a chronic illness has both positive and negative effects (Fisman, Wolf, Ellison, & Freeman, 2000). However, the negative effects are rather immediate while the positive effects are obvious later on, i.e. in adolescence or even adulthood (Rossiter & Sharpe, 2001). Most of the siblings to children with disabilities or chronic illnesses cope well with the situation (Cuskelly & Gunn, 2006; Levy-Wasser & Katz, 2004), while a minority are at risk of developing severe adjustment difficulties (Fisman et al., 2000; Giallo, Gavida-Payne, Minett, & Kapoor, 2012; Pit-ten Cate & Loots, 2000; Rossiter & Sharpe, 2001) such as problems in school, decreased self-esteem and social stigma (Williams, 1997). Additionally, they can experience that their needs are neglected and put aside by their strained parents, which can further negatively affect positive development (Abell & Gecas, 1997).

There are few studies conducted concerning the situation for siblings in regards to gender, age and birth number (Levy-Wasser & Katz, 2004) and more studies are needed to be able to see the full picture (Nielsen et al., 2012). However, what these studies reveal so far is that sisters, mostly older sisters (Olsen et al., 1999), are more involved in the care of their siblings than the brothers (Dyson, 2010) and that this responsibility is more or less permanent (Lobato, 1990). This responsibility may include: baby-sitting, being a significant other/friend, or helping the child with special needs to socialize outside the home (Floyd et al., 2009; Gallagher, Powell, & Rhodes, 2006). Older sisters also feel and express more worries regarding the sibling situation and well-being (Guse & Harvey, 2010) which taken together with this responsibility constitutes a significant stressor for the older sisters (Olsen et al., 1999). This can lead to severe adjustment problems and psychosocial ill health both in the short- and long-term (Thompson, Curtner, & O'Rear, 1994).

The teenage period, characterized by personal and emotional conflicts leading to the urge to separate from the rest of the family and aspects concerning identity and conformity, is especially important for the growing child. The period itself is demanding and stressful and often negatively affects the ability to cope with the stressful situation of living with and caring for a sibling with a disability or chronic illness (Newman & Newman, 1997). Relations with friends can become complicated since most of them lack insight into how to live with a sibling with special needs, and can even lead to teasing or bullying (Barr & McLeod, 2010). Contacts and relationships can also be affected negatively when living with a sibling with special needs (Gamble & Woulbroun, 1993). Further, teenagers often experience a limited ability to co-operate with the rest of the family and thereby limited possibilities to express thoughts and feelings for their sick sibling and adjust to the stressful situation. This, in turn, can lead to feelings of guilt (Opperman & Alant, 2003) at the same time as conflicts within the family increase during this period and also the emotional distance (Coch, Fischer, & Dawson, 2007), which constitutes an increased risk for severe psychosocial problems in the child.

Mediating factors for psychosocial problems in these siblings are an open dialogue with parents where they are allowed to express both positive and negative feelings towards the situation of the family (Siegel & Silverstein, 1994). Also, support groups for siblings where the child can freely express and discuss their thoughts and feelings have been shown to positively affect the child's well-being and self-esteem (Smith & Perry, 2005), especially if the parents also attend the support groups with the child (Burke & Montgomery, 2001). A short-break outside the home for the child with a disability or chronic illness can also affect the siblings positively (Welch et al., 2012) by reducing stress (Sherman, 1995) and increased self-esteem (Evans, Jones, & Mansell, 2001; Lobato & Kao, 2002).

In summary, having a sibling with a disability or chronic illness constitutes a risk for severe psychosocial ill-health in the child. For older sisters who additionally often have permanent caretaking responsibilities for their sick sibling, in contrast to the brothers, that risk are probably even higher, especially during the vulnerable teenage period. To further illuminate and target the situation and the psychosocial well-being for older sisters of children with disabilities or chronic illnesses in research studies and in clinical work should be of interest, not only because of the immediate risk-factors for these girls during childhood but also to protect these vulnerable girls from future psychosocial suffering, even in adulthood.

*Ulrika Hallberg* Sociologist/Associate Professor
